# Agenesis of the gallbladder and cystic duct

**DOI:** 10.1590/S1516-31802002000600008

**Published:** 2002-11-01

**Authors:** Jaques Waisberg, Paulo Engler Pinto, Paula Regina Gusson, Paola Rossini Fasano, Antônio Cláudio de Godoy

**Keywords:** Gallbladder, Gallbladder diseases, Cholecystectomy, Vesícula biliar, Doenças da vesícula biliar, Colecistectomia

## Abstract

Agenesis of the gallbladder and cystic duct is a rare anomaly that is usually asymptomatic. The patient may present symptoms characteristic of cholelithiasis. Its surgical confirmation requires careful dissection of the common bile duct and intraoperative cholangiography or ultrasonography to be performed, to exclude the possibility of an ectopic gallbladder. The authors describe two cases of this unusual affection and comment on its clinical, pathophysiological and diagnostic aspects.

## RESUMO

A agenesia de vesícula biliar e do ducto cístico é anomalia rara e habitualmente assinto- mática. O doente pode apresentar sintomas característicos de colecistopatia. A confirma-ção cirúrgica exige dissecção cuidadosa do hepatocolédoco e realização de colangiografia ou ultra-sonografia intra-operatórias para excluir vesícula biliar ectópica. Os autores descrevem dois casos desta inusitada afecção e comentam seus aspectos clínicos, pato-fisiológicos e diagnósticos.

## INTRODUCTION

Agenesis of the gallbladder is a rare congenital condition characterized by the absence of the gallbladder and cystic duct in adult patients, without other malformations of the internal or external hepatic bile ducts.^1^ Its presumed incidence rate is from 0.09 to 0.016% in necropsies. Cases diagnosed through operations present a female predominance of 3:1, although in autopsies the proportions are equal between the sexes. Usually, adults are asymptomatic, although they may present abdominal pain and/or jaundice.^2^

## CASE REPORT

### Case 1

A 68-year-old white woman who had had colicky pains in the upper abdomen for 10 years, accompanied by nausea and vomiting and intolerance towards fatty foods, was referred to our service. Her physical examination was uneventful. Two ultrasound scans of the upper abdomen were performed, neither of which identified the gallbladder, and this suggested the presence of a sclerotic or atrophied gallbladder. The patient was operated on, and upon making the inventory of the cavity, the gallbladder was not found. Intraoperative cholangiography performed via puncturing the bile duct using a needle was normal, without identifying the gallbladder. The patient was discharged from hospital without having had any intercurrence and remains asymptomatic three years after the operation.

### Case 2

**Figure 1:**
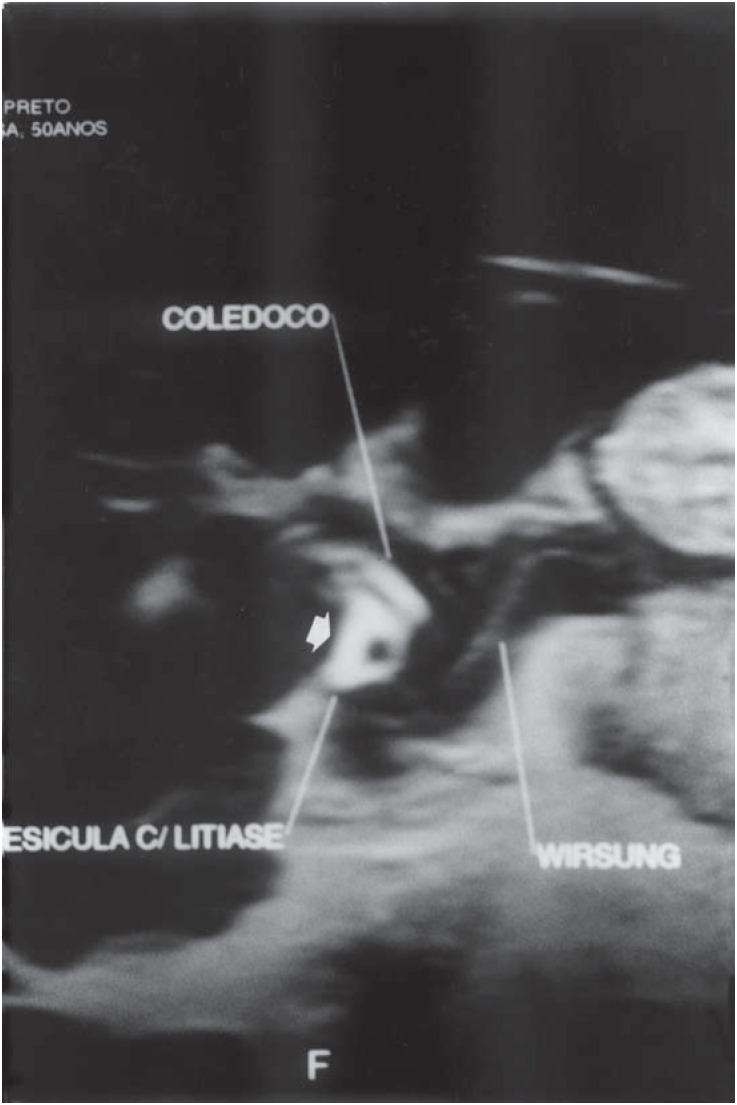
Cholangioresonance with an image interpreted at that time as "gallbladder with lithiasis" (white arrow) (case 2).

A 50-year-old white woman who reported having had episodes of severe colicky pain in the upper right quadrant over the past two years was seen in a consultation at another hospital. Her physical examination was normal. Ultrasonography revealed "a gallbladder of normal shape and dimensions, with normal thickness and multiple internal images" and an "abnormal" location for the gallbladder, close to the gastric antrum, in the interpretation made at that time. The patient underwent laparoscopy and the gallbladder was not found. On this occasion, intraoperative cholangiography was not performed. She presented a further five episodes of abdominal pain that were similar to the initial picture, and the cholangioresonance report revealed "a retro-antral and retro-duodenal gallbladder with reduced dimensions and lithiasis" (Figure 1), according to the interpretation made at that time. The patient underwent another laparoscopy in which no gallbladder was encountered, with the operation being converted into laparotomy. Once again, exploration of the peritoneal cavity and intraoperative cholangiography did not locate the gallbladder. The patient continued to have symptoms and was transferred to our service. Duodenography was performed and did not reveal any duodenal diverticulum, but despite the previous surgical findings, an image obtained from cholangioresonance was considered strongly suggestive of the presence of an ectopic gallbladder. The patient was submitted to exploratory laparotomy and the external bile duct was dissected from the confluence of the hepatic duct to the duodenum.

Intraoperative ultrasonography and intraoperative cholangiography via puncturing the bile duct using a fine needle did not reveal a gallbladder or a cystic duct. The patient was released from hospital without having had any intercurrence and remains asymptomatic two years after the last operation.

## COMMENTS

Embryologically, the development of the liver and gallbladder system starts around the third week of gestation, when the primordial liver, designated the hepatic diverticulum, is formed as an outgrowth of the endodermis in the distal part of the anterior intestine.^1,3^ As the diverticulum grows, its connection with the intestine narrows to form the external hepatic bile duct. A small ventral invagination grows in this area of narrowing and gradually forms a vacuole that becomes the gallbladder and cystic duct. A failure of this invagination results in agenesis of the gallbladder and cystic duct without any associated atresia of the external hepatic bile duct.^4^

Agenesis of the gallbladder found in adults has rare associations with other malforma- tions.^2,4^ The majority of adult patients with agenesis of the gallbladder are aged between 36 and 46 years.^2^ There is no characteristic symptomatology associated with this anomaly.^2^ It has been reported that approximately 23% of the patients with agenesis of the gallbladder will develop symptoms suggestive of bile tract disease at some time in their lives,^2^ and that in around 25% to 50% of them, calculi will be found inside the dilated common bile duct.^3,5^

It is believed that there is a pathophysiological similarity between agenesis of the gallbladder and the dilatation of the hepatic bile duct that may occur after cholecystectomy.^2^ In agenesis, the hepatic bile duct may substitute for the absent gallbladder by becoming dilated and taking on the function of bile stor- age.^2^ In this situation, the presence of dyskinesia of the bile tract, elevation of the basal pressure of the Oddi sphincter, cholestasis or infection of the bile ducts may provoke the onset of a clinical condition and/or lithiasis of the common bile duct, especially when two or more of these events occur in association.^2,4,5^

Usually, the diagnosis is established during the operation,^3,5^ as happened with the patients we treated. The diagnosis is not considered to be accurate until laparotomy or autopsy has been performed because the radiological investigative methods for gallbladder diseases present sensitivity of less than 100% for the identification of the organ.^5^ Thus, a diagnosis of agenesis of the gallbladder can only be confirmed by surgical exploration and intraoperative cholangiography or ultrasonography, fundamental for excluding the possibility of hypoplasia or an aberrant localization of the gallbladder or, under other circumstances, by examination during a necropsy.^2^ The surgeon needs to be alert to the risk of bile duct fistula occurring secondary to intraoperative cholangiography performed by puncturing the main bile duct, which is usually found to be normal in these cases.

During the surgical intervention, to be sure that the gallbladder is really absent, the common bile duct must be identified along its whole length from the confluence of the right and left hepatic ducts to the duode-num.^2,5^ The surgical dissection of the extrahepatic bile duct must be done carefully to avoid direct lesions of the bile duct structures or their vascularization, situations that can favor cicatricial stenosis.

The two patients in this report, as well as the great majority of the cases described in the literature, became asymptomatic after the surgical procedures. It has been speculated that lysis of adherences localized in the upper right quadrant of the abdomen or at periportal sites contributes towards controlling the clinical condition.^1^

Once it has been discovered via laparotomy that the gallbladder appears to be absent, we believe it is the surgeon's responsibility to prove agenesis of the organ. For this, intraoperative cholangiography or ultrasonography must be performed and the external hepatic bile duct must be carefully identified along its entire length in an attempt to localize the organ that is apparently absent.
